# Canine Distemper Epizootic among Red Foxes, Italy, 2009

**DOI:** 10.3201/eid1612.100579

**Published:** 2010-12

**Authors:** Vito Martella, Alessandro Bianchi, Irene Bertoletti, Luca Pedrotti, Alessandro Gugiatti, Alessia Catella, Paolo Cordioli, Maria S. Lucente, Gabriella Elia, Canio Buonavoglia

**Affiliations:** Author affiliations: Università di Bari, Bari, Italy (V. Martella, M.S. Lucente, G. Elia, C. Buonavoglia);; Istituto Zooprofilattico Sperimentale della Lombardia e dell'Emilia Romagna, Sezione di Sondrio, Italy (A. Bianchi, I. Bertoletti);; Consorzio del Parco Nazionale dello Stelvio, Bormio, Sondrio (L. Pedrotti, A. Gugiatti);; Istituto Zooprofilattico Sperimentale della Lombardia e dell'Emilia Romagna, Sezione di Brescia, Italy (A. Catella, P. Cordioli)

**Keywords:** Canine distemper virus, fox, red foxes, Italy, *Vulpes vulpes*, H gene, viruses, letter

**To the Editor:** Canine distemper virus (CDV) is an enveloped, single-stranded, negative RNA virus of the family *Paramyxoviridae,* genus *Morbillivirus* (*1*). The host range for CDV is broad, and infection has been found in several mammalian species of the families Canidae, Mustelidae, Procyonidae, Ursidae, and Viverridae (*2*).

Stelvio National Park (SNP) encompasses 1,333 km^2^ of protected land in Italy and covers 2 regions (Lombardia and Trentino Alto Adige); the Lombardia section of the park covers the northern part of Sondrio Province (Valtellina). SNP is surrounded by other parks (Schweitzer National Park, Adamello, and Adamello-Brenta) to form a large protected area (2,500 km^2^) in the heart of the Alps mountains. Within SNP, the terrestrial mammals that are susceptible to CDV include red foxes (*Vulpes vulpes*), stoats (*Mustela erminea*), weasels (*Mustela nivalis*), pine martens (*Martes martes*), beech martens (*Martes foina*), badgers (*Meles meles*), and bears (*Ursus arctos*).

In August 2009, three young red foxes were captured in Valtellina (Sondrio), Lombardia, Italy, within the southwestern borders of SNP. The animals showed canine distemper–like signs (e.g., prostration, altered behavior, and conjunctivitis), and CDV infection was confirmed by quantitative reverse transcription–PCR of pooled organs (*3*). In September and October 2009, another 2 young foxes were captured and found to be positive for CDV. From September on, at least 30 foxes with altered behavior were seen near human habitations and facilities in SNP; 10 were captured. In the same period, infected foxes were also reported from Engadina, Switzerland, at the northern and western borders of SNP. In February 2010, two symptomatic foxes were euthanized in Grosotto, 50 km south of where the initial cases were identified. The epizootic appeared to have originated from the eastern regions of Italy (Trentino Alto Adige, and Veneto), where CDV infection had been reported in red foxes and badgers since August 2006 (*4*) (Figure). A large CDV epidemic in foxes in southern Bavaria in 2008 has also been described, thus suggesting spread of the virus throughout the Alps area (*5*).

**Figure:**
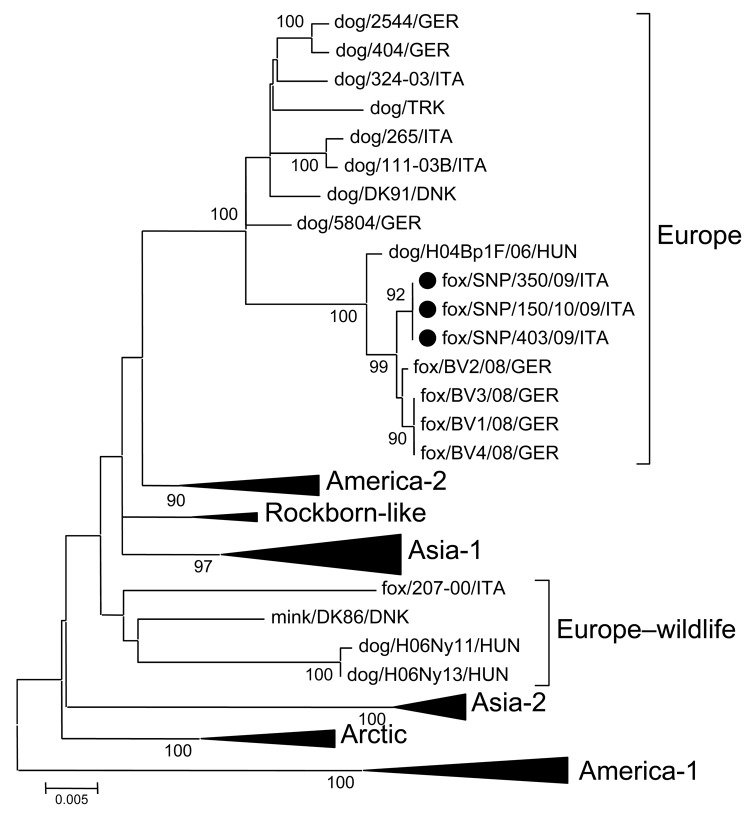
Phylogenetic tree showing the genetic relationships among selected canine distemper virus strains of various lineages and generated by using the full-length nucleotide sequence of the hemagglutinin gene. The tree branches including viruses not from Europe were collapsed (triangles). Full circles indicate the canine distemper strains identified in foxes from Stelvio National Park, Italy. The neighbor-joining tree was generated by using the Kimura 2-parameter distance correction, and statistical support was provided by bootstrapping >1,000 replicates, using the software package MEGA4 (www.megasoftware.net). Scale bar indicates nucleotide substitutions per site.

Reverse transcription–PCR genotyping of the hemagglutinin (H) gene (*6*) identified 15 CDV strains, which were analyzed and characterized as European genotypes. The full-length H gene of the CDV strains was determined (GenBank accession no. HM120874). Sequence analysis of the H gene indicated that the fox CDV strains were highly related to each other (>99.9% nt and 100% aa identity), to the CDV strains identified in foxes in southern Bavaria 2008 (>99.7% nt and 99.3% aa identity; accession nos. FI416336–FI416338), and to a canine strain identified in Hungary during 2005–2006 (99.4% nt and 99.2% aa identity; accession no. DQ889177).

During the CDV epizootic in SNP in 2009, cases of CDV in 3 domestic dogs living within the borders of the park were also reported. Because vaccination against CDV is a core recommendation for dogs, most dogs are expected to be vaccinated and protected; population immunity is high enough to keep CDV infection under control, and only sporadic cases occur (*7*). Accordingly, the reported CDV cases in dogs were more likely a spillover event caused by the high pressure of CDV infection in SNP wildlife. In addition to foxes, badgers in the same area were also reported to have canine distemper–like disease. These findings are consistent with spread of a multihost epizootic, in which foxes likely played a major role in CDV amplification and diffusion because of their social behavior during reproductive season and because of the wide geographic range over which juveniles migrate during the dispersion period.

Serologic investigations for CDV in some fox populations in Europe have identified antibody prevalence rates of 4%–26.4% (*8*), suggesting that CDV circulates in foxes in Europe, but these investigations did not examine spatial and temporal variations in CDV activity. Clues for understanding the pattern of CDV disease in wildlife have been provided by structured surveillance of wild canids living in Yellowstone National Park, USA. Yearly fluctuations in CDV seroprevalence with evidence of multihost outbreaks in distinct years, contemporaneously affecting different animal species, have been noted. Cycles of CDV epizootics that swept through the animals in the park were associated with low pup survival rates (*9*).

In SNP, most foxes captured during the epizootic were juveniles. We have no information on the prevalence rates of CDV-specific antibodies in SNP foxes before the epizootic. However, CDV disease had not been reported in the SNP for at least 10 years, and no animal with CDV infection had been identified in a 2004–2005 survey of red foxes in SNP (*10*). Similarly, no evidence for CDV infection had been found in carnivores in Trentino Alto Adige during 2001–2002 (*10*). Accordingly, one can assume that the population immunity in SNP foxes (and in other susceptible hosts) was low.

Adequately controlling CDV infection in wildlife in Europe is difficult. It requires concerted transnational actions, including effective surveillance and prompt gathering and dissemination of information.
